# Bibliographical

**Published:** 1886-11

**Authors:** 


### BIBLIOGRAPHICAL.

### A Practical Treatise on Mechanical Dentistry.

By Joseph Richardson, M.D., D.D.S., Late Emeritus Professor of the Principles of Prosthetic Dentistry in the Indiana Dental College; Formerly Professor of Mechanical Dentistry and Metallurgy in the Ohio College of Dental Surgery. Fourth edition, revised and enlarged, with four hundred and fifty-eight illustrations.

This work has just been issued from the publishing house of P. Blakiston, Son & Co. The make-up of the book is better than that of the former editions, and this, everyone will concede, is saying a good deal.

As to the character and value of the additions made to this edition, it seems almost superfluous to say anything to those acquainted with the former editions of the work. The former editions fully showed the ability and disposition of the author to keep well abreast with the times.

Within the last ten years there have been greater advances made in supplying artificial substitutes than ever before in the same length of time, indeed in some respects entirely new methods have been devised, introduced, and largely adopted in the profession. All this has been gathered and put in good and most available form in this issue.

In addition to this, old methods and processes have been improved and rendered more simple and available. All this has been faithfully recorded.

Dr. Richardson's natural endowments and acquired abilities, and opportunities as a teacher, writer, and investigator have fitted him in an eminent degree for the preparation of this work.

As a text book for the student, and a reference book for the practitioner, it has no equal in this country.

### New Journals.

We have just received the prospectus of *The Dental Review*, a monthly journal, the first number of which is to be issued November 15th, 1886, and thereafter regularly at the same date of each month. The prospectus proposes and promises large things, and judging by the names proposed as contributors, the promises will be fulfilled. If *The Review* succeeds in inducing an increased number of careful readers in our profession, it will have accomplished a very desirable and praiseworthy object. *The Review* is published in Chicago by W. T. Keener. The editor is evidently a modest man, thus far at least, for his name does not appear in the prospectus. Doubtless he will make his appearance ere long.

Another, *The Western Dental Journal*, is to make its appearance at the beginning of 1887. Kansas City has the honor of being the place of its nativity. It is to be issued monthly, and will have for its editor Dr. J. D. Patterson, D.D.S., and he for his associates, Dr. C. K. Hungerford, D.D.S., and A. H. Thompson, D.D.S. R. I. Pearson & Co., publishers.

The multiplication of periodicals is an encouraging feature in the progress of our profession. Every new journal develops additional new writers, and this is a stimulus to those who have been writing in the past years. In one aspect, at least, our publications have not been sufficient for the needs of the profession, which is clearly shown by the fact that at almost every dental association embracing any considerable number of persons, instruments, appliances, and modes of practice are brought to light, of which no record has been made; and, further than this, the principles upon which our practice is established have not been so presented and discussed that all, or even a majority understand them. Competition in investigating and recording leads to greater accuracy and the clearer illucidation of truth. Then may the largest possible number lend a helping hand, and keep the pages of our journals overflowing all the while.

